# Exocrine pancreas function is impaired in adult relatives of patients with type 1 diabetes

**DOI:** 10.1007/s00592-021-01819-2

**Published:** 2021-11-15

**Authors:** Anna Giovenzana, Federica Vecchio, Federica Cugnata, Alessandro Nonis, Alessandra Mandelli, Angela Stabilini, Benedetta Allegra Mazzi, Maurizio De Pellegrin, Andrea Laurenzi, Riccardo Bonfanti, Manuela Battaglia, Emanuele Bosi, Alessandra Petrelli

**Affiliations:** 1 San Raffaele Diabetes Research Institute, IRCCS Ospedale San Raffaele, Via Olgettina 58, 20132 Milan, Italy; 2 University Centre of Statistics for Biomedical Sciences (CUSSB), Vita-Salute San Raffaele University, Milan, Italy; 3 Department of Internal Medicine, Diabetology, Endocrinology and Metabolism, IRCCS Ospedale San Raffaele, Milan, Italy; 4 Pediatric Department, IRCCS Ospedale San Raffaele, Milan, Italy; 5 Vita-Salute San Raffaele University, Via Olgettina, 60, 20132 Milan, Italy; 6 Immuno-Hematology and Transfusion Medicine (ITMS), IRCCS Ospedale San Raffaele, Milan, Italy; 7 Pediatric Orthopedic Unit, IRCCS Ospedale San Raffaele, Milan, Italy; 8 Cochin Institute, Paris Descartes University, Paris, France; 9 Telethon Foundation, Milan, Italy

**Keywords:** Type 1 diabetes, Exocrine pancreas, P-amylase, Pre-symptomatic T1D

## Abstract

**Aims:**

Alterations of the exocrine pancreas have been reported in type 1 diabetes, but their contribution to the pathogenesis of the disease is poorly understood. Here, we investigated markers of exocrine pancreas dysfunction in individuals at-risk of developing type 1 diabetes.

**Methods:**

Serum P-amylase and lipase levels were assessed in samples obtained from healthy controls, patients with new onset type 1 diabetes, relatives participating to the TrialNet Pathway to Prevention who were, at blood collection, autoantibody negative or positive for a single autoantibody (low-risk individuals), and positive for multiple autoantibodies (high-risk individuals). Linear mixed models were adopted to estimate variation of pancreatic enzymes among the groups and to evaluate the influence of high-risk HLA genotypes and residual beta cell function on exocrine pancreas function.

**Results:**

In adults, but not children, reduced levels of P-amylase and lipase were shown in at-risk individuals, including (for P-amylase levels only) those at low-risk, and in T1Dnew. Furthermore, while high-risk HLA genotypes negatively affected P-amylase levels in autoantibody negative adult individuals, fasting C-peptide levels did not correlate with pancreatic enzyme levels.

**Conclusions:**

Exocrine pancreas dysfunction precedes the onset of type 1 diabetes in adult at-risk individuals and may be unrelated to fasting C-peptide levels.

**Supplementary Information:**

The online version contains supplementary material available at 10.1007/s00592-021-01819-2.

## Introduction

Type 1 diabetes (T1D) has been recently reconsidered as a disease of the whole pancreas, rather than exclusively characterized by self-reactive T cell-mediated beta cell destruction. In fact, consistent evidence now suggests that the exocrine component of the pancreas, mainly composed of acinar cells that produce digestive enzymes, is also involved in the disease process leading to beta cell loss.

Data corroborating this hypothesis are structural, immunopathological and functional. Pancreas weight and volume are reduced in patients with overt type 1 diabetes as well as at-risk individuals, including relatives with and without islet autoantibodies, suggesting that pancreas remodeling occurs before and after seroconversion and may be genetically determined [1–7]. Immune cells are abundant both in the islets and the exocrine compartment [8–13], and autoantibodies targeting exocrine pancreas-derived proteins were described in at-risk individuals as well as in patients with type 1 diabetes [14]. The reduced pancreatic mass, accompanied by immune cell infiltration, results in a subclinical exocrine functional impairment, characterized by an overall reduction of exocrine pancreatic enzymes, with trypsinogen, lipase and fecal elastase (FE-1) levels already decreased in at-risk individuals [15–18].

The heterogeneous clinical phenotype observed in patients with type 1 diabetes led investigators to propose the use of the ‘endotype model’, originally applied to the asthma field [19], also in type 1 diabetes. This model is based on the assumption that different mechanisms underlie the pathogenesis of the disease and can explain, at least to some extent, the variety of clinical phenotypes [20]. Here, we hypothesized that exocrine pancreas dysfunction contributes to type 1 diabetes heterogeneity; therefore, in this study, we attempted to clarify how pancreatic enzyme abnormalities associate with age, sex, high-risk HLA genotypes, islet autoantibodies status and fasting C-peptide in individuals at various stages (low-risk, high-risk, clinical onset) of progression to type 1 diabetes.

Understanding the patterns of exocrine pancreas pathophysiology in at-risk, pre-symptomatic individuals can contribute to the understanding of type 1 diabetes heterogeneity and underlying endotypes.

## Methods

### Subjects

Subjects participating in this study were enrolled at Ospedale San Raffaele (OSR), Milan (Italy) from December 2010 to February 2019. In this study, 3 groups of subjects were enrolled. Group 1: relatives of patients with T1D were participants to the TN01 TrialNet Pathway to Prevention Study (formerly Natural History Study) [21]. Based on their islet autoantibody profile at study entry, relatives were recruited as follows: autoantibody negative (AAb−, *n* = 276), subjects positive for single (AAb1 + , *n* = 74) or multiple (2 or more) autoantibodies (AAb ≥ 2 + , *n* = 50). The risk of progression to clinical type 1 diabetes is negligible in those negative, around 15% in those with a single autoantibody (low-risk), and more than 80% in those with multiple autoantibodies (high-risk) in the following 15 years [22, 23]. A fraction of at-risk subjects, equal to 14.6% (107 subjects out of 732), was followed longitudinally in the TN01, and multiple samples obtained from these individuals were used in this study (referred to as repeated measures). The total number of samples was 1092 from 732 subjects within 598 families. Group 2: patients with new onset type 1 diabetes (T1Dnew, *n* = 86) according to the American Diabetes Association criteria [24] were enrolled either at the Pediatric or General Medicine, Diabetes and Endocrinology Departments of the Ospedale San Raffaele, Milan, Italy. Blood samples were withdrawn within 3 months from diagnosis. In patients presenting with diabetic ketoacidosis (DKA), blood samples were collected at least five days after hospital admission to rule out potential confounding factors affecting pancreatic enzyme levels [25]. Group 3: healthy non-diabetic controls (HC, *n* = 246) with no family history for type 1 diabetes undergoing surgery for congenital diseases (i.e., flatfeet or hallux valgus) at the OSR Orthopedic Department. Subjects with current infection or missing data were excluded from the study. Detailed characteristics of participants are shown in Table 1.

**Table 1:** Donor characteristics

	HC *N* = 247	AAb− *N* = 343	AAb1 + *N* = 208	AAb ≥ 2 + *N* = 208	T1Dnew *N* = 86
Age (years), median (range)	16.97 (1.45–48.08)	12.78 (1.11–46.03)	18.06 (3.31–51.04)	15.53 (4.32–45.56)	13.39 (3.89–47.62)
Sex					
Female, *n*(%)	116 (46.96%)	191 (55.69%)	101 (48.56%)	106 (50.96%)	35 (40.7%)
Male, *n*(%)	131 (53.04%)	152 (44.31%)	107 (51.44%)	102 (49.04%)	51 (59.3%)
*Number of Autoantibodies*	n/a				
0, *n*(%)		343 (100%)			1 (1.16%)
1, *n*(%)			208 (100%)		12 (13.95%)
2, *n*(%)				91 (43.75%)	15 (17.44%)
3, *n*(%)				74 (35.58%)	38 (44.19%)
4, *n*(%)				38 (18.27%)	20 (23.26%)
5, *n*(%)				5 (2.40%)	0
Type of autoantibody	n/a				
GAD65, *n*(%)			167 (80.29%)	197 (94.71%)	72 (83.72%)
ZnT8, *n*(%)			0	133 (63.94%)	67 (77.91%)
IA2, *n*(%)			6 (2.88%)	70 (33.65%)	54 (62.79%)
ICA, *n*(%)			1 (0.48%)	110 (52.88%)	0
IAA, *n*(%)			33 (15.87%)	71 (34.13%)	43 (50%)
Pancreatic Amylase (U/L)	25 (5–111)	22 (4–93)	23 (2–85)	23 (0–93)	16.5 (7–128)
Lipase (U/L)	26 (11–153)	27 (12–183)	26 (9–81)	24 (9–83)	23 (12–350)
Fasting c-peptide, median (ng/ml)	n/a	*N* = 64 1.61(0.595–3.695)	*N* = 159 1.55(0.435–4.35)	*N* = 188 1.455(0.375–3.25)	*N* = 79 0.41(0–2.8)
HLA (DR3 or DR4), *n*(%)	n/a	*N* = 97 67 (69.07%)	*N* = 206 136 (66.02%)	*N* = 201 153 (76.12%)	n/a

All characteristics are shown for the total 1092 observations (732 subjects and 598 families, range number of observations per subject = 1–13). HC, Healthy controls; AAb−, Autoantibody negative; AAb1 + , Positive for 1 islet autoantibody; AAb ≥ 2 + , Positive for 2 or more islet autoantibodies; T1Dnew, New onset type 1 diabetes patients. *Data are presented as absolute %, mean* ± *SD, or median (first quartile, third quartile)*

### Study approval

The study was approved by the Ospedale San Raffaele (OSR) Ethics Committee (IRB#TIGET004-DRI003). First-degree relatives of T1D patients were enrolled in the T1D TrialNet Pathway to Prevention Trial (TN01) at OSR (IRB# NHPROT32803-TN01). Peripheral blood was collected for mechanistic studies (approved by the TrialNet Ancillary Studies Subcommittee). All participants (or parents for pediatric individuals) provided written informed consent. Participants over the age of 12 signed an additional study assent form.

### Blood tests

Serum P-amylase, lipase and C-peptide concentrations were measured in the Ospedale San Raffaele central laboratory by automated methods, as follows. Serum P-amylase levels were measured using the enzymatic colorimetric method after inhibition of human salivary α-amylase (Cobas C, Roche/Hitachi, Roche Diagnostics GmbH, Mannheim, Germany); serum lipase levels using the enzymatic colorimetric method (Cobas C, Roche/Hitachi, Roche Diagnostics GmbH, Mannheim, Germany); fasting serum C-peptide levels using Electrochemiluminescent Assay, CLIA, on automatic Instrumentation Cobas 8000 (Roche Diagnostics GmbH, Mannheim, Germany). Reference ranges for the above enzymes are: P-amylase, 0–20 U/L (0–24 months), 9–35 U/L (2–18 years) subjects, 13–53 U/L (> 18 years); lipase: 0–60 U/L. The following islet autoantibodies were measured in all enrolled participants except HC: islet cell antibodies (ICA), anti-IA2, anti-GAD, anti-insulin (IAA) and anti-ZnT8. Measurements were performed according to study protocol [21]. ICA were not routinely measured in T1Dnew. HLA genotypes were performed in an European Federation for Immunogenetics (EFI) accredited laboratory using the HISTO SPOT SSO System (BAG Health Care GmbH, Germany). DRB1*03 (DR3) and DRB1*04 (DR4) alleles are considered high-risk HLA genotypes for the development of type 1 diabetes [26].

### Statistical analysis

Linear mixed-effects models (LME) were employed to estimate the longitudinal trend of the amylase and the lipase and evaluate the differences among groups, since the data consist of repeated measurements of the same subjects with some subjects belonging to the same family. Random effects were accordingly defined, nested by family and by subject. In order to meet the assumptions of normality of the model, the square root transformation was applied to the amylase and the logarithmic transformation was applied to the lipase. Both linear and quadratic terms for age at sample collection were included in the model to account for the nonlinear trajectories of amylase and lipase over time. In the models, we also accounted for the possible effect of sex and the disease-groups (HC, AAb−, AAb1 + or AAb ≥ 2 + and T1Dnew) at sample collection, for the interaction effect between the sex and the disease-groups and for the interaction effect between the age and the disease-groups. Backward selection procedures were applied to select the most parsimonious model. Post-hoc analysis after LME was performed, considering all the pairwise comparisons of disease-groups at a fixed age. *p*-values were adjusted using Bonferroni’s correction. LME models were also employed to evaluate the effect of high-risk HLA genotypes and fasting C-peptide on amylase and lipase. These effects were evaluated separately and accounting for the possible effects of age, sex and disease-groups and the interactions with these variables. All statistical analyses were performed using R 3.6.3 (http://www.R-project.org/) and the significance threshold was set at 0.05.

## Results

### Serum levels of P-amylase and lipase are decreased in adult relatives of patients with type 1 diabetes, but not in children

**Fig. 1:**
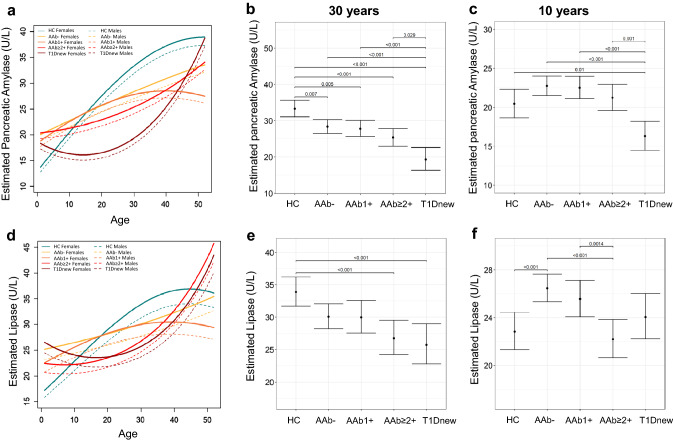
Reduction of serum amylase and lipase occurs in adult relatives of type 1 diabetes but not in children. **a** Final linear mixed-effects model for predicting serum P-amylase levels based on disease-groups, age and sex as well as interactions among variables are shown. **b**–**c** Pairwise post-hoc comparisons among groups at fixed age for estimated level of P-amylase in female subjects. **d** Final linear mixed-effects model for predicting serum lipase levels on the basis of the disease-groups, age and sex as well as interactions with them are shown. **e**–**f** Pairwise post-hoc comparisons among groups at fixed age for estimated level of lipase in female subjects. Only significative comparisons *p*-values are shown in the graph

Serum P-amylase and lipase levels were evaluated in relation to age and sex in HC, relatives of patients with type 1 diabetes (i.e., AAb−, AAb1 + or AAb ≥ 2 +) and T1Dnew. Table 1 shows characteristics of participants including all 1092 observations from 732 subjects. The linear mixed-effects model showed that P-amylase levels increase with age in all groups although with different trends (Fig. 1a and Table S1). In HC, a significant positive linear effect of age is evident. Low-risk relatives (i.e., AAb− and AAb1 + individuals), although presenting a lower slope than HC, also showed increased P-amylase levels with age. In T1Dnew, P-amylase levels decreased during childhood and early adolescence, starting to rise only after the age of 15. Of note, high-risk individuals (AAb ≥ 2 +) showed a relatively flat curve until their 20 s, with an intermediate pattern between low-risk relatives and T1Dnew. Although within the normal range, in adults (Fig. 1b showing the status at age 30) estimated serum P-amylase levels were significantly reduced in at-risk individuals, already before seroconversion, and in T1Dnew patients. This was not observed in children, where the significant reduction was evident only in T1Dnew (Fig. 1c showing the status at age 10).

Age-dependent levels of lipase showed a similar trend compared to P-amylase, but differed in that HC have a less steep curve and subjects with multiple autoantibodies overlap with T1Dnew patients (Fig. 1d and Table S2). Serum lipase levels in adults (30 years; Fig. 1e and Figure S1b left panel), but not in children (10 years; Fig. 1f and Fig. S1b right panel), were found significantly reduced in relatives with multiple autoantibodies and in T1Dnew patients compared to HCs. No statistical differences were evident between adult low-risk relatives and HCs (AAb− vs. HC, *p* = 0.090; AAb1 + vs. HC, *p* = 0.209) (Fig. 1e). Unexpectedly, lipase levels were increased in AAb− pediatric individuals compared to HCs, but declined in AAb ≥ 2 + subjects.

With regard to sex, males showed significantly lower levels of both P-amylase and lipase compared to females within all groups (Fig. S1).

Overall, these data indicate that exocrine pancreas function is impaired in adults, but not in children, already during pre-symptomatic stages of type 1 diabetes. Moreover, in the case of P-amylase, function impairment is detectable already prior to islet autoantibody seroconversion, suggesting a possible genetic predisposition to exocrine dysfunction.

### High-risk HLA genotypes negatively affect P-amylase levels of AAb1+ adult individuals

The effect of high-risk HLA genotypes DR3 and/or DR4 on the exocrine pancreas function was tested in at-risk individuals. The presence of high-risk HLA genotypes has a negative effect on P-amylase (Table 2) in AAb1+ individuals only (AAb1+ × Age x HLA, *p*-value = 0.024), with a trend observed in AAb ≥ 2+ subjects (Ab POS ≥ 2+ × Age x HLA, *p*-value = 0.099), and no effect in AAb− (Ab POS ≥ 2+ × Age x HLA, *p*-value = 0.773). Notably, the effect of high-risk HLA in AAb1+ relatives was greater with increasing age (AAb1+ × Age, Ab POS ≥ 2+ × Age, *p*-value < 0.001), suggesting that the high-risk HLA genotypes are associated with lower P-amylase levels in adults more than in children. No effect of high-risk HLA genotypes was observed on lipase levels (data not shown).

**Table 2:** Effect of high-risk HLA genotypes on P-amylase levels

Parameter	Estimate	SEr	*p*-value
Intercept	4.317	0.116	< 0.001
AAb− x Age	0.017	0.011	0.117
AAb1+ × Age	0.035	0.007	< 0.001
Ab POS ≥ 2+ × Age	0.032	0.009	< 0.001
AAb− x Age x HLA	0.003	0.011	0.773
AAb1+ × Age x HLA	−0.016	0.007	0.024
Ab POS ≥ 2+ × Age x HLA	−0.014	0.009	0.099

### Fasting C-peptide levels do not correlate with pancreatic enzymes reduction

To test the hypothesis that exocrine pancreas dysfunction is influenced by the loss of insulin secretion during the pre-symptomatic stage of type 1 diabetes, we evaluated the effect of fasting C-peptide on P-amylase and lipase levels, accounting for the possible effects of age, sex and disease-groups and the interactions with these variables. After backward selection, the final models did not include fasting C-peptide levels (data not shown) indicating lack of relationship between the two variables.

## Discussion

Exocrine pancreas dysfunction is commonly accepted as a feature of type 1 diabetes, as evidence from multiple independent studies have shown decreased levels of pancreatic enzymes [15–18, 27–30]. In this study, we demonstrate that impaired exocrine function, resulting in lower levels of circulating P-amylase and lipase, can be detected long before the onset of clinical symptoms in at-risk adult individuals, but not in children. It can be postulated that the exocrine pancreas is able to better counteract type 1 diabetes-associated pathogenetic destruction during infancy and early adolescence than in adult age, possibly thanks to the development and regeneration ability of the pancreas in childhood [31]. This would lead to stable P-amylase levels during the pre-symptomatic stages of type 1 diabetes as observed at the age of 10, while declining thereafter as shown at the age of 30. Since the study population was not randomly selected as it is part of a large T1D prevention trial, it cannot be excluded that, when subjects were aware of their autoantibody status, their lifestyle habits changed in such a way as to affect P-amylase and lipase levels. Furthermore, although this is an observational study in which subjects were selected retrospectively, with a non-negligible selection bias, our analysis allowed to control for a number of confounding factors. To strengthen these results, a validation cohort would be needed.

Reduced levels of FE-1 [16], serum trypsinogen [17] and lipase [18] have been reported in children at-risk of developing type 1 diabetes. In our analysis, we have taken into account the effect of multiple parameters (such as age, sex and number of autoantibodies) on P-amylase and lipase levels. The different pattern of exocrine pancreas dysfunction observed between children and adults may explain why, in another study [18], pancreatic enzymes were not found reduced in individuals with low-risk of developing type 1 diabetes. However, it should be noted that a limitation of our study is the lack of body mass index (BMI) and pubertal status data, which may affect pancreatic enzyme levels [32]. Although serum P-amylase and lipase levels are not considered sensitive biomarkers of exocrine pancreas function [33], our data are consistent with previous reports showing that exocrine pancreatic enzymes are a serological biomarker for T1D staging.

The lack of relationship between P-amylase with fasting C-peptide levels suggests that the counterregulatory mechanisms against the progression of type 1 diabetes in younger age are more effective for the exocrine, as reflected by stable P-amylase, than the endocrine pancreas, as reflected by declining C-peptide. On the other hand, this divergence is unlikely to persist with advancing age and disease progression, as indicated by the recently reported correlation between P-amylase and C-peptide in adult new onset and long-standing type 1 diabetes [29]. However, it should be considered that fasting C-peptide, although considered a reasonably good indicator of endogenous insulin secretion [34], it is not the best way to estimate residual beta cell function, thus leaving still uncertain whether exocrine and endocrine compartments of the pancreas are interdependent in the natural history of type 1 diabetes.

The evidence that low-risk adult relatives display reduced P-amylase levels suggests that the genetic background may influence the impairment of exocrine pancreas function. However, with regard to HLA, we show that high-risk genotypes impact exocrine dysfunction only in adult single autoantibody-positive individuals. It is possible that increasing sample size may help clarify the effect of HLA genotypes in multiple autoantibody-positive subjects. However, since other non-HLA genes may be equally involved in exocrine pancreas dysfunction, additional studies are needed to more comprehensively address the role of the genetics.

There is growing evidence that, behind the clinical phenotype, there are different mechanisms contributing to the pathogenesis of type 1 diabetes, thus highlighting the need to unveil disease endotypes [20]. Besides the heterogeneity of type 1 diabetes in terms of age at disease onset, genetic susceptibility, decline of residual beta cell function and pancreas-specific immune cell infiltrate, here we provide evidence that exocrine pancreas dysfunction occurs in at-risk adult individuals, possibly reflecting an endotype with a low progression rate. So far, the abnormalities of exocrine pancreatic function have been considered subclinical; however, in the perspective of a new trait of endotype heterogeneity, the possible clinical impact of exocrine pancreatic dysfunction in type 1 diabetes should be studied more in depth in the future.

## Conclusion

In conclusion, our findings provide evidence that the exocrine pancreas dysfunction detected during the pre-symptomatic stages of type 1 diabetes show different patterns of progression according to age, possibly reflecting a further trait of endotype heterogeneity.

## Supplementary Information

Below is the link to the electronic supplementary material.

Supplementary file1 (DOCX 145 kb)

## Data Availability

The data supporting the findings of this study are available within the article [and/or] its supplementary materials.

## Abbreviations

AAb−: Autoantibody negative
AAb1 +: Positive for 1 islet autoantibody
AAb ≥ 2 +: Positive for 2 or more islet autoantibodies
BMI: Body Mass Index
DKA: Diabetic ketoacidosis
DR3: DRB1*03
DR4: DRB1*04
FE-1: Fecal elastase-1
GAD: Glutamic acid decarboxylase
HC: Healthy controls
HLA: Human Leukocyte Antigens
IA2: IA‐2 molecule (IA‐2A)
IAA: Insulin autoantibodies
ICA: Cytoplasmic islet cell antibodies
LME: Linear mixed model
OSR: Ospedale San Raffaele
P-amylase: Pancreatic amylase
T1D: Type 1 diabetes
T1Dnew: New onset type 1 diabetes patients
TN01: TrialNet Pathway to Prevention Study
ZnT8: Zinc transporter isoform 8
