# Supplementary material for: Exocrine pancreas function is impaired in adult relatives of patients with type 1 diabetes
Source: Acta Diabetol. 2021 Nov 15;59(4):473–9. doi: 10.1007/s00592-021-01819-2 (PMC8917021; doi:10.1007/s00592-021-01819-2)

**Supplementary Tables**

**Table S1. Analysis of amylase variation according to age within control and disease groups.**

| \| **Parameter** \| **Estimate** \| **Std.Error** \| **p-value** \| \| --- \| --- \| --- \| --- \| \| Intercept \| 3.604 \| 0.293 \| <0.001 \| \| Gender (Male) \| -0.127 \| 0.061 \| 0.040 \| \| AAb- \| 0.838 \| 0.322 \| 0.010 \| \| AAb1+ \| 0.679 \| 0.329 \| 0.040 \| \| AAb≥2+ \| 0.901 \| 0.363 \| 0.014 \| \| T1Dnew \| 0.718 \| 0.452 \| 0.115 \| \| HC x Age \| 0.102 \| 0.027 \| <0.001 \| \| AAb- x Age \| 0.034 \| 0.016 \| 0.032 \| \| AAb1+ x Age \| 0.053 \| 0.016 \| 0.001 \| \| AAb≥2+ x Age \| 0.007 \| 0.023 \| 0.762 \| \| T1Dnew x Age \| -0.044 \| 0.037 \| 0.238 \| \| HC x Age^2^ \| -0.001 \| 0.001 \| 0.077 \| \| AAb- x Age^2^ \| -0.0002 \| 0.000 \| 0.634 \| \| AAb1+ x Age^2^ \| -0.001 \| 0.000 \| 0.034 \| \| AAb≥2+ x Age^2^ \| 0.0004 \| 0.001 \| 0.430 \| \| T1Dnew x Age^2^ \| 0.002 \| 0.001 \| 0.048 \| |
| --- | --- | --- | --- | --- | --- | --- | --- | --- | --- | --- | --- | --- | --- | --- | --- | --- | --- | --- | --- | --- | --- | --- | --- | --- | --- | --- | --- | --- | --- | --- | --- | --- | --- | --- | --- | --- | --- | --- | --- | --- | --- | --- | --- | --- | --- | --- | --- | --- | --- | --- | --- | --- | --- | --- | --- | --- | --- | --- | --- | --- | --- | --- | --- | --- | --- | --- | --- | --- |
|  |

Final linear mixed-effects model for predicting serum P-amylase levels based on disease-groups, age and sex as well as interactions among variables are shown, after backward variable selection. Age is expressed in linear and quadratic terms.

| **Table S2. Analysis of lipase variation accordingly to age within control and disease groups.** |
| --- |
| \| **Parameter** \| **Estimate** \| **Std.Error** \| **p-value** \| \| --- \| --- \| --- \| --- \| \| Intercept \| 2.810 \| 0.098 \| <0.001 \| \| Gender (Male) \| -0.081 \| 0.021 \| <0.001 \| \| AAb- \| 0.410 \| 0.111 \| <0.001 \| \| AAb1+ \| 0.288 \| 0.120 \| 0.017 \| \| AAb≥2+ \| 0.304 \| 0.137 \| 0.027 \| \| T1Dnew \| 0.483 \| 0.153 \| 0.002 \| \| HC x Age \| 0.036 \| 0.009 \| <0.001 \| \| AAb- x Age \| 0.005 \| 0.006 \| 0.390 \| \| AAb1+ x Age \| 0.016 \| 0.007 \| 0.024 \| \| AAb≥2+ x Age \| -0.005 \| 0.010 \| 0.626 \| \| T1Dnew x Age \| -0.016 \| 0.013 \| 0.206 \| \| HC x Age^2^ \| -0.0004 \| 0.0002 \| 0.031 \| \| AAb- x Age^2^ \| 0.00003 \| 0.0001 \| 0.844 \| \| AAb1+ x Age^2^ \| -0.0002 \| 0.0001 \| 0.125 \| \| AAb≥2+ x Age^2^ \| 0.0004 \| 0.0002 \| 0.085 \| \| T1Dnew x Age^2^ \| 0.001 \| 0.0003 \| 0.072 \| |

Final linear mixed-effects model for predicting serum lipase levels on the basis of the disease-groups. Sex and age (linear and quadratic terms) as well as interactions with them are shown.

**Figure S1.** **Reduction of serum amylase and lipase occurs in adult relatives of T1D but not in children (male data).** (a) Pairwise post-hoc comparisons among groups at fixed age, 30 years (left panel), 10 years (right panel) for estimated level of P-amylase in male subjects; (b) Pairwise post-hoc comparisons among groups at fixed age, 30 years (left panel), 10 years (right panel) for estimated level of lipase in male subjects. Only significative comparisons’ p-values are shown in the graph.


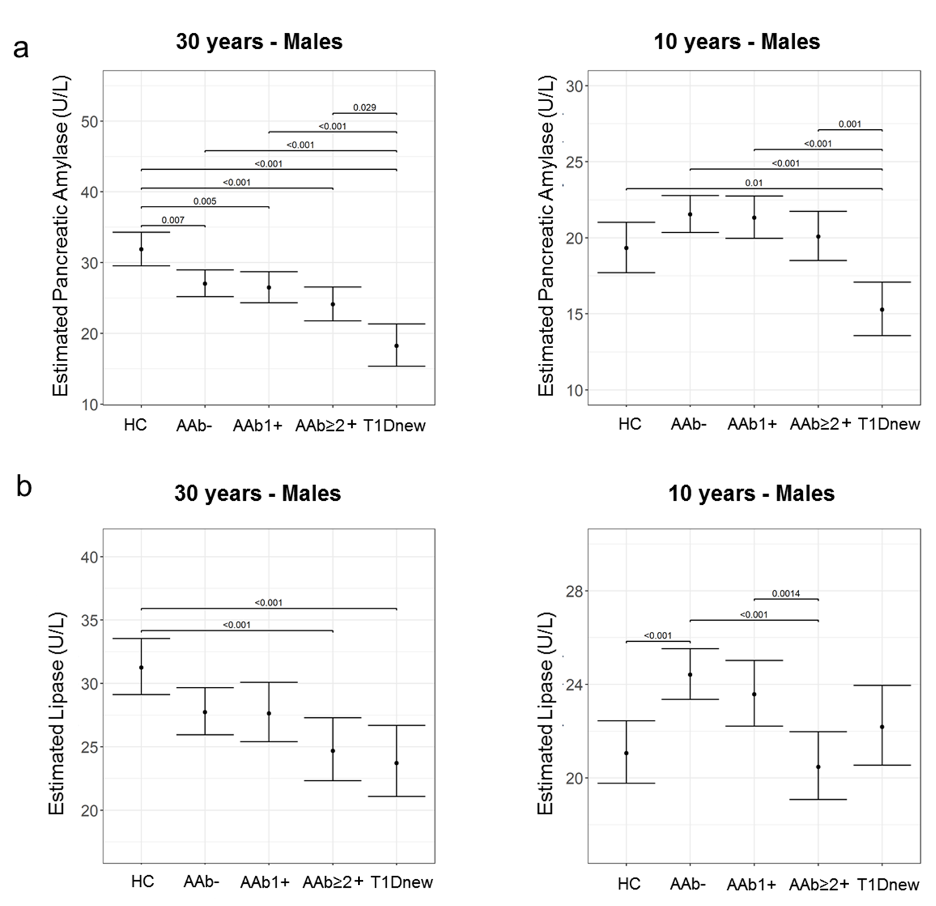

Supplement: Supplementary file 1 — Supplementary file1 (DOCX 145 kb) [file 592_2021_1819_MOESM1_ESM.docx]
